# A Systematic Review of the Gonadotoxicity of Osteosarcoma and Ewing’s Sarcoma Chemotherapies in Postpubertal Females and Males

**DOI:** 10.1089/jayao.2023.0185

**Published:** 2024-08-02

**Authors:** Susanna Weidlinger, Satu Graber, Irina Bratschi, Janna Pape, Attila Kollár, Tanya Karrer, Michael von Wolff

**Affiliations:** ^1^ Division of Gynecological Endocrinology and Reproductive Medicine, University Women’s Hospital, Inselspital Bern, University of Bern, Bern, Switzerland.; ^2^ Department of Medical Oncology, Inselspital Bern, University of Bern, Bern, Switzerland.; ^3^ Medical Library, University Library Bern, University of Bern, Bern, Switzerland.

**Keywords:** FertiTOX, FertiPROTEKT, osteosarcoma, Ewing’s sarcoma, fertility, anti-mullerian hormone, amenorrhea, sperm count, gonadotoxicity, chemotherapy, radiotherapy

## Abstract

Data on gonadotoxicity of chemotherapies are essential to better counsel young females and males about the risk of infertility and to better indicate fertility preservation measures before cancer therapies. However, such data have not recently been reviewed for bone cancer. Therefore, a systematic literature search was conducted considering papers published since 2000. This study is part of the FertiTOX^®^ project, which aims to improve the lack of data regarding gonadotoxicity of cancer therapies to enable more accurate counseling regarding fertility preservation. Only relapse-free women and men were included. Gonadotoxic therapy-induced suspected infertility was defined as very low anti-mullerian hormone, high gonadotropin concentration, amenorrhea, oligomenorrhea, azoospermia, or oligozoospermia. The quality of the individual studies was assessed using the Newcastle–Ottawa Scale (NOS). In total, 11 out of 831 studies were included in the review. Suspected infertility was found in 10/190 (5.1%, range 0%–66%) of female patients with osteosarcoma (six studies), in 24/46 (52.2%, range 46%–100%) of male patients with osteosarcoma (three studies), in 18/138 (13.0%, range 3%–18%) of female patients with Ewing’s sarcoma (three studies), and in 34/38 (89.5%) of male patients with Ewing’s sarcoma (one study). A risk calculation in relation to specific chemotherapies was not possible. Risk of suspected infertility tends to be higher in Ewing’s sarcoma in which all patients received chemotherapies with alkylating agents. Two of the 11 included studies received a high NOS quality score, whereas the remaining nine studies received a low quality score, mainly because of the lack of a comparator group. Published data are too limited for precise estimation of the gonadotoxicity. However, data indicate clinically relevant risk for infertility, supporting counseling patients before chemotherapy about fertility preservation measures.

## Introduction

Since the first three milestones in fertility preservation had been reached, such as the first birth after transplantation of cryopreserved ovarian tissue,^1^ the introduction of stimulation protocols that allow oocyte collection within 2 weeks^2^ and vitrification of oocytes,^3^ fertility preservation measures have been introduced in most countries. Fertility preservation has now been accepted and defined as an important element to be considered before cancer treatments in females and males.^4–9^

One of the most important criteria that has to be met to recommend fertility-preserving measures is the actual risk of infertility because of the gonadotoxicity of the applied cancer therapy. However, data on the gonadotoxicity of therapies of different forms of cancer and the numerous cancer treatment protocols are mostly very limited. Accordingly, indications for or against fertility-preserving measures are not well defined, which, on the one hand, carries the risk of overtreatment of patients with fertility preservation measures, imposing unnecessary medical risks and burdens to patients as well as unnecessarily postponing cancer therapies. On the other hand, it carries the risk of undertreatment with fertility-preserving measures, which, in the case of infertility after surviving cancer, can substantially impair the quality of life.^10^

Osteosarcoma and Ewing’s sarcoma are two types of cancer with a high incidence in adolescents and young adults with still limited survival rates. In osteosarcoma, survival rates have not substantially increased since the introduction of chemotherapies in the 20th century. Currently, the 5-year survival rate of osteosarcoma is 76% for localized cancer and 64% for regional and 24% for distant spread of cancer.^11^

In Ewing’s sarcoma, new treatment protocols gradually increased survival rates, but overall survival rates are still relatively low with 82% for localized cancer and 71% for regional and 39% for distant spread of cancer.^11^

Owing to strong chemotherapies, fertility is still a major issue in bone cancer disease.^8^ European guidelines state that the rate of treatment-induced amenorrhea in survivors of osteosarcoma and Ewing’s sarcoma treated with anthracycline- and cyclophosphamide-based chemotherapy regimens with or without radiotherapy ranges between 3% and 25%^12,13^ and that predisposing factors for a higher risk of permanent amenorrhea are older age and use of high-dose chemotherapy and radiotherapy.^12^ However, this statement is based on only one large Italian registry analysis,^12^ including patients treated between 1983 and 2006 and another systematic review on osteosarcoma,^13^ including only three studies with a total of 29 survivors treated. A recent and systematic review to specifically review the gonadotoxicity of bone cancer is still missing. Even though the gonadotoxicity of specific chemotherapies can be estimated using risk calculators, which are even available online (www.oncofertilityrisk.com), the risk of infertility also needs to be provided on a disease-specific basis. The specific chemotherapy and its total dosage may not be defined at the time of fertility preservation counseling, the chemotherapy may even be adjusted during the course of therapy, and the accuracy of the prediction still needs to be validated.

We, therefore, set up a series of systematic reviews (www.fertitox.com)^14,15^ to close the gap of data regarding gonadotoxicity of cancer therapies to better counsel young adults about treatment-related risk of infertility and the necessity to undergo fertility preservation measures.

As published data are only available for osteosarcoma and Ewing’s sarcoma, the most common bone sarcomas, but not on chondrosarcoma and fibrosarcoma, this systematic review analyses only these two cancer types. To evaluate the impact of the chemotherapies on fertility, only relapse-free cases were included. Prepubertal individuals were excluded as fertility could hardly be analyzed if chemotherapy was applied at a very young age. This study is part of the FertiTOX^®^ project, which aims to improve the lack of data regarding gonadotoxicity of cancer therapies to enable more accurate counseling regarding fertility preservation.

## Materials and Methods

### Protocol registration

The study protocol was registered at the international Prospective Register of Systematic Reviews, PROSPERO (Registry number 331654). The Preferred Reporting Items for Systematic Reviews and Meta-Analyses^16^ was used.

### Information sources and search methods

To identify all potentially relevant documents on the topic, complex literature searches were designed and executed for the following information sources: MEDLINE, Embase, and Cochrane Library.

An initial search strategy was developed in MEDLINE by a medical information specialist and tested against a list of core references to see if they were included in the search result. After refinement and consultation, complex search strategies were set up for each information source based on database-specific controlled vocabulary (thesaurus terms/subject headings) and text words. Synonyms, acronyms, and similar terms were included in the text word search. The only limit that was applied to all searched databases was the year of publication from 2000 to the present.

All searches were run on August 11, 2022.

The search concepts included were (1) four types of sarcoma (chondrosarcoma, fibrosarcoma, osteosarcoma, and Ewing’s sarcoma), (2) two types of cancer therapies (chemotherapy and radiotherapy), and (3) gonadotoxic effects or influences on fertility parameters. Synonyms, acronyms, and similar terms were used for all concepts in the text word search, as well as the respective thesaurus terms.

Studies concerning exclusively animals were excluded from the searches in MEDLINE and Embase by using a double-negative search strategy based on the “humans only” filters by Ovid.

The detailed final search strategies are presented as Supplementary Data S1.

In addition to electronic database searching, reference lists and bibliographies from relevant publications were checked for relevant studies.

### Study selection process

All identified citations were imported into EndNote, and duplicates were removed. The screening of titles and abstracts was performed by S.G., I.B., and S.W. and tested against the inclusion criteria (Table 1) with the support of the software Covidence (www.covidence.org). Cancer treatments were evaluated regarding their clinically relevant gonadotoxicity. Clinically relevant gonadotoxicity was defined as suspected infertility, defined by the criteria shown in Table 2.

**Table 1.** Inclusion and Exclusion Criteria

Inclusion criteria
Any original papers with information on tumor type, tumor therapy, and fertility results (fertility parameters as shown in Table 2)
Papers in which fertility data were analyzed and described separately for the different cancer types and for females and males
Exclusion criteria
Patients with prepubertal status or >40 years of age at the time of potentially gonadotoxic therapy
Patients with cancer relapse and palliative treatment
Patients with stem-cell transplantation
Females with radiotherapy of the pelvis
Papers with <40% subject participation in the evaluation of reproductive markers

**Table 2.** Definition of Suspected Infertility

Females
Menstrual cycle disorders (amenorrhea, oligomenorrhea)
Gonadotropins (follicle-stimulating hormone; luteinizing hormone) above the normal range
Anti-mullerian hormone below the detection limit
Premature ovarian insufficiency
Males
Significant reduction in sperm quality (azoospermia, oligozoospermia)

### Quality assessment

The quality of the individual studies was assessed using the Newcastle–Ottawa Scale (NOS).^17^ The assessment system is based on a “star system,” according to which each study is assessed based on three aspects: the selection of the study groups, the comparability of the groups, and the coverage of the exposure or outcome of interest. Rating: good quality: 3 or 4 stars in selection domain AND 1 or 2 stars in comparability domain AND 2 or 3 stars in outcome/exposure domain; fair quality: 2 stars in selection domain AND 1 or 2 stars in comparability domain AND 2 or 3 stars in outcome/exposure domain; poor quality: 0 or 1 star in selection domain OR 0 stars in comparability domain OR 0 or 1 stars in outcome/exposure domain.

All included studies were reviewed by S.G., I.B., and S.W. to independently assess the risk of bias. Disagreements were resolved by consensus. With the exception of the study by Bishop et al. 2020^18^ and Mörse et al. 2016^19^ in which the methodological quality was rated good, corresponding to a low risk of bias, the methodological quality of the remaining nine studies^12,20–27^ was rated low, mainly because of the lack of the comparison group (Table 3).

**Table 3.** Newcastle–Ottawa Quality Assessment Form for Cohort Studies

	Selection				Comparability		Outcome					
Study	Representa-tiveness of exposed cohort	Selection of nonexposed cohort	Ascertainment of exposure	Outcome of interest not present at the start of the study	Comparability of cohorts in main factors	Comparability of cohorts in additional factors	Assessment of outcome	Sufficient length of follow-up for outcomes to occur	Adequacy of follow-up of cohorts	Total	Quality assessment	Comments
Bishop^18^	**★**	**★**	**★**	–	**★**	**★**	**★**	**★**	**★**	8/9	Good	
Mörse^19^	**★**	–	**★**	**★**	**★**	**★**	**★**	**★**	**★**	8/9	Good	
Gupta^23^	**★**	–	**★**	**★**	–	–	**★**	**★**	**★**	6/9	Poor	No comparison group
Raciborska^25^	**★**	–	**★**	–	–	–	**★**	**★**	–	4/9	Poor	No comparison group
Kenney^24^	**★**	–	**★**	–	–	–	**★**	**★**	–	4/9	Poor	No comparison group
Rendtorff^26^	**★**	–	**★**	–	–	–	**★**	**★**	–	4/9	Poor	No comparison group
Longhi^12^	**★**	–	**★**	–	–	–	**★**	**★**	**★**	5/9	Poor	No comparison group
Durrieu^22^	**★**	–	**★**	–	–	–	**★**	**★**	**★**	5/9	Poor	No comparison group
Bacci^21^	**★**	–	**★**	–	–	–	**★**	**★**	**★**	5/9	Poor	No comparison group
Wikström^27^	**★**	–	**★**	–	–	–	**★**	**★**	**★**	5/9	Poor	No comparison group
Bacci^20^	**★**	–	**★**	–	–	–	**★**	**★**	**★**	5/9	Poor	No comparison group

## Results

### Study characteristics

**FIG. 1.**
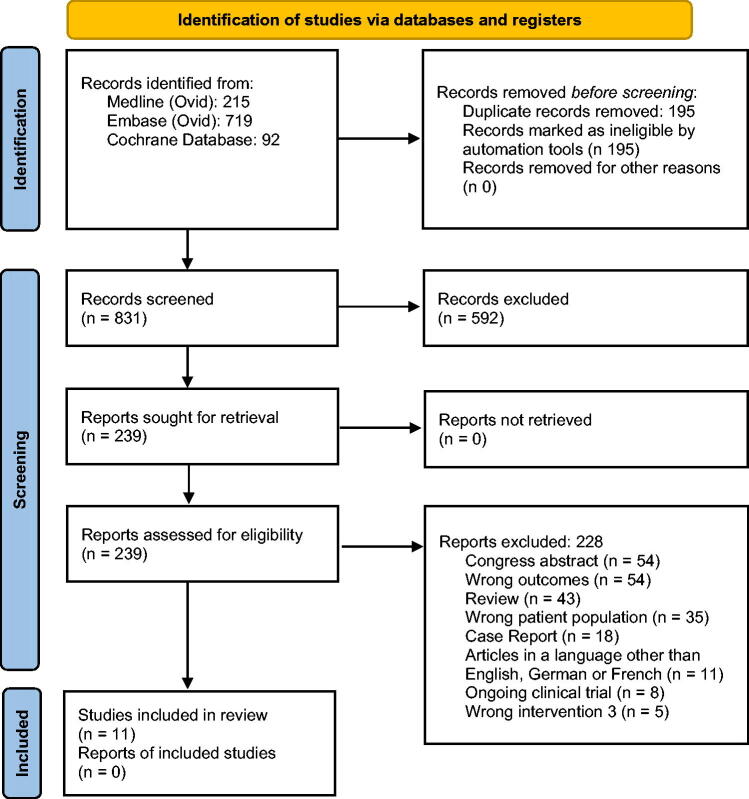
The Preferred Reporting Items for Systematic Reviews and Meta-Analyses flow diagram.

In total, 11 out of 831 studies were included in the review (Table 4, Fig. 1). All studies were registry analyses or observational studies. The reported outcome parameters regarding fertility were mainly the menstrual status (amenorrhea or oligomenorrhea) as well as anti-mullerian hormone (AMH) and follicle-stimulating hormone concentration indicating premature ovarian insufficiency and ejaculate quality (azoospermia or oligozoospermia) not allowing or substantially reducing the chance of spontaneous conception. The number of participants with osteosarcoma or Ewing’s sarcoma per study varied in females from 1 to 154 and in males from 3 to 38 included patients. In some studies, certain parameters such as age at diagnosis/therapy and length of follow-up were calculated for the total number of patients evaluated in the study rather than for the subpopulation of patients with osteosarcoma and Ewing’s sarcoma separately (see comments in Table 4). Accordingly, these information might be slightly different for the subset of patients included in the analysis.

**Table 4.** Characteristics of the Included Studies

First author, year of publication	Identification of participants of interest (cases, females, pubertal status at the time of diagnosis/therapy)	Number of participants of interest	Age of participants of interest at the time of diagnosis/therapy	Diagnosis/treatment period	Follow-up after diagnosis/treatment, length in years (range)	Type of chemotherapy	Reported outcome parameter and outcome	% suspected infertility	Comments
Osteosarcoma, females									
Bacci et al.^20^	164 patients with OS, 54 females, 26 postpubertal, in 1 cancer center in Italy	**26** females	<14y (*n* = 75)	1986–1989	Median 11.5y (10–13)*	- With alkylating agents: 43/164 = 26%*	Menstrual status: 0 with amenorrhea	0/26 = 0%	*Calculated for 164 patients with OS
			>14y: (*n* = 89)*			- Without alkylating agents: 121/164 = 74%*			
Wikström et al.^27^	10 females with OS, 5 postpubertal, in 1 cancer center in Finland	**5** females	Median 12.9y (6.4–15.2)**	1984–1999	Median 2.8y (1.5–4.9)***	- With alkylating agents: 5/5 = 100%	Occurrence of POI, menstrual status and FSH concentration: 1 with diagnosis of POI, 1 with irregular menstrual status	2/5 = 40%	** Calculated for 10 females with OS
						- Without alkylating agents: 0/5 = 0%			*** Calculated for 5 postpubertal females with OS
Durrieu et al.^22^	16 females with cancer, 1 postpubertal with OS, in 1 cancer center in France	**1** female	23y	1991	4y	- With alkylating agents: 1/1 = 100%-Without alkylating agents: 0/1 = 0%	Menstrual status: 0 with amenorrhea	0/1 = 0%	
Longhi et al.^12^	883 patients with OS, 573 alive, 231 females, 207 evaluated for infertility, 154 postpubertal, in 6 cancer centers in Italy	**154** females	Median 15y (1–40)****	1983–2006	Median 11.8y (3.4–27.3)*****	- With alkylating agents: 756/883 = 26%****	Menstrual status: 6 with amenorrhea	6/154 = 4%	**** Calculated for 883 patients with OS
						- Without alkylating agents: 127/883 = 14%****			*****Calculated for 573 patients with OS alive
Gupta et al.^23^	16 females with cancer, 3 postpubertal with OS, in 1 cancer center in Canada	**3** females	Median 15.7y (13.2–16.0)	2010–2011	Median 1.8y (1.3–2.0)	- With alkylating agents: 0/3 = 0%	Menstrual status and AMH concentration: 1 with amenorrhea, 1 with AMH below detection limit	2/3 = 66%	
						- Without alkylating agents: 3/3 = 100%			
Mörse et al.^19^	21 females with cancer, 4 with OS, 2 postpubertal, 1 without recurrence, in 1 cancer center in Sweden	**1** female	14.2y	2006 (year of ethical approval)	2.8y (after treatment completion); 3.4y (after diagnosis)	- With alkylating agents: 0/1 = 0%	AMH and FSH concentration: 0 with abnormal results	0/1 = 0%	
						- Without alkylating agents: 1/1 = 100%			
Osteosarcoma, males									
Rendtorff et al.^26^	77 males with cancer, 3 with OS and unknown pubertal status, in 1 cancer center in Germany	**3** males	Median 15y (11–16)	1980–2009	Median 8y (4–18)	No information	Semen analysis: 2 with azoospermia, 1 with oligozoospermia	3/3 = 100%	3 of 3 eligible males consented to semen analysis (100%).
Kenney et al.^24^	6 postpubertal males with OS, in 2 U.S. cancer centers	**6** males	Median 16.5y (15–17)		Median 4y (2–8)	- With alkylating agents: 6/6 = 100%	Semen analysis: 2 with azoospermia, 2 with oligozoospermia	4/6 = 66%	Only 6 of 15 eligible males consented to semen analysis (40%).
						- Without alkylating agents: 0/6 = 0%			
Bishop et al.^18^	117 patients with OS and unknown pubertal status, 63 males, 37 with semen analysis, in 1 U.S. cancer center	**37** males	Median: 13.8y (range 3.2–23.6)^×^	1964–2002	≥10y	- With alkylating agents: 109/117 = 93%^×^	Semen analysis: 17 with azoospermia or oligozoospermia	17/37 = 46%	Only 37 of 63 eligible males consented to semen analysis (59%). ^×^Calculated for 117 patients with OS
						- Without alkylating agents: 8/117 = 7%^×^			
Ewing’s sarcoma, females									
Bacci et al.^21^	402 patients with ES, 82 female long-term survivors, 34 postpubertal, in 1 Italian cancer center	**34** females	<40 (inclusion criterion)	1972–1992	Mean 17.7y (10–30)°	- With alkylating agents: 34/34 = 100%	Menstrual status: 1 with amenorrhea	1/34 = 3%	°Calculated for 402 patients with ES
						- Without alkylating agents: 0/34 = 0%			
Longhi et al.^12^	543 patients with ES, 323 alive, 123 females, 99 evaluated for infertility, 63 postpubertal, in 6 cancer centers in Italy (Italian Sarcoma Group)	**93** females	Median 15y (1–40)°°	1983–2006	Median 11.8y (0.8–27.8)°°°	- With alkylating agents: 93/93 = 100%	Menstrual status: 15 with amenorrhea	15/93 = 16%	°°Calculated for 99 patients with ES evaluated for infertility
						- Without alkylating agents: 0/93 = 0%			°°°Calculated for 323 patients with ES alive
Raciborska et al.^25^	27 female patients with ES, 12 without pelvic radiation or stem cell transplantation, 11 postpubertal, in 5 Polish cancer centers	**11** females	Median 13.9y (range 2.8–17.7)°°°°	2000–2012	Median 5.7y (range 2.0–13.6)°°°°°	- With alkylating agents: 11/11 = 100%	Occurrence of POI, FSH concentration and menstrual status: 2 with POI (1 with FSH above normal range and amenorrhea 1 with FSH above normal range)	2/11 = 18%	°°°°Calculated for 12 female patients without pelvic radiation or stem cell transplantation
						- Without alkylating agents: 0/11 = 0%			°°°°°Calculated for 27 female patients with ES
Ewing’s sarcoma, males									
Bishop et al.^18^	90 patients with ES and unknown pubertal status, 53 males, 38 with semen analysis, in 1 U.S. center	**38** males	Median: 13.3y (range 1.7–24.8)˜	1964–2002	≥10y	- With alkylating agents: 90/90 = 100%˜	Semen analysis: 34 with azoospermia or oligozoospermia	34/38 = 90%	Only 38 of 53 eligible males consented to semen analysis (72%).
						- Without alkylating agents: 0/90 = 0%˜			30% of 90 patients with ES received pelvic radiation.
									˜Calculated for 90 patients with ES

AMH, anti-mullerian hormone; ES, Ewing’s sarcoma; FSH, follicle-stimulating hormone; OS, osteosarcoma; POI, premature ovarian insufficiency.

### Data analysis in patients with osteosarcoma

Suspected infertility was found in 10/190 (5.3%, range 0%–66%) of female patients with osteosarcoma (six studies)^12,19,20,22,23,27^ and in 24/46 (52.2%, range 46%–100%) of male patients with osteosarcoma (three studies).^18,24,26^ Around 40% of females with osteosarcoma and around 90% of males received chemotherapies with alkylants (Table 3). The rate of suspected infertility varied considerably. Overall rates seemed to be higher in males than in females. However, it needs to be noted that not all men accepted semen analysis, potentially leading to some bias in the selection of patients.

### Data analysis in patients with Ewing’s sarcoma

Suspected infertility was found in 18/138 (13.0%, range 3%–18%) of female patients with Ewing’s sarcoma (three studies)^12,21,25^ and in 34/38 (89.5%) of male patients with Ewing’s Sarcoma (one study)^18^ (Table 3). All patients with Ewing’s sarcoma received chemotherapies with alkylants. The rate of suspected infertility also varied considerably in patients with Ewing’s sarcoma. As in osteosarcoma, rates seemed to be higher in males than in females. However, as in the osteosarcoma group, not all men accepted semen analysis, and furthermore, only one male study was included in the analysis.

## Discussion

The purpose of the systematic review was to summarize data on the gonadotoxicity of osteosarcoma and Ewinǵs sarcoma chemotherapies to better counsel females and males about the risk of infertility and the need to perform fertility preservation measures before cancer therapy.

Our study showed that in osteosarcoma, the risk for suspected infertility is around 5.3% in females and 52.2% in males. In Ewing’s sarcoma, it is around 13.0% in females and 89.5% in males.

The strength of our study is that it is based on clinically relevant infertility parameters such as very low AMH or high gonadotropin concentrations, amenorrhea, oligomenorrhea, azoospermia, or oligozoospermia, indicating reduced chances of spontaneous conception, which we summarized under the term “suspected infertility.” Another strength is that only postpubertal patients with known pubertal status and without pelvic radiation (in females) and patients without bone marrow transplantation were included in our analysis, which allowed us to evaluate specifically the gonadotoxicity of chemotherapies.

However, both strengths could also be defined as weaknesses. The chosen fertility markers indicate some disruption of the hypothalamic–pituitary–gonadal axis and thus suspected infertility but not definite infertility. Furthermore, owing to the exclusion of prepubertal patients and those with pelvic radiation and bone marrow transplantation, our study does not cover the whole spectrum of cancer therapies in this specific patient population. Another weakness is that in the majority of studies (9/11), it is not known if the selected markers were affected because of the gonadotoxic therapies or if fertility was already reduced before chemotherapy.

However, owing to the limited data available and the heterogeneity of the fertility-related outcome parameters described in the included studies, we decided to summarize the mentioned markers under the term “suspected infertility” and to evaluate the papers accordingly. Hence, this term can be seen as the best possible option to draw at least some conclusions regarding the gonadotoxicity of the chemotherapies used in patients with osteosarcoma and Ewing’s sarcoma.

The very limited and heterogenous data might also be a reason why almost no other systematic reviews have been published so far addressing the gonadotoxicity of bone cancer therapies. Only one systematic review has been published in 2017.^13^ It included only three studies with a total of 29 survivors treated. Another systematic review was published in 2020,^28^ but this review only included three studies with pregnancy and childbirth as outcome parameters.

Our study demonstrates variability of data regarding the risk of infertility after chemotherapy. However, in spite of the variability, the available data indicate a clinically relevant infertility risk. The risk in Ewing’s sarcoma seems to be higher than in osteosarcoma, probably because of a higher proportion of patients receiving chemotherapies with alkylants. In line with this, the rate of suspected infertility was higher in male than in female patients with osteosarcoma as males received more frequently alkylants. Alkylants, especially in combination with cisplatin, seem to be highly gonadotoxic as shown in males.^20,24,29^ However, owing to the high variability of our data, with a broad range of suspected infertility of 0%–66% in female and 46%–100% in male patients with osteosarcoma and of 3%–18% in female and 90% in male patients with Ewing’s sarcoma, our findings need to be taken with great care.

The same applies to our finding that the risk of infertility seems to be higher in males than in females. In males, we can expect a substantial bias in the data as only a limited number of males performed a semen analysis. It can be assumed that the proportion of included males who had not fathered a child when the study was performed is higher than those had not.

We tried to reduce this bias by excluding papers with <40% of subject participation in the evaluation of reproductive markers. However, 40% of participation is a very low cutoff level, which still might have caused substantial bias. But choosing a higher level would have led to exclusion of most, if not of all studies in males.

Our study did not allow us to review systematically the impact of factors such as intensified chemotherapies or age on fertility. These factors were only analyzed sporadically in a very few studies.

Yonemoto et al. found out that the intensity of chemotherapies has an impact on fertility.^30^ They analyzed the fertility rate, defined as offspring of 29 married male patients who had received chemotherapy for osteosarcoma and compared these couples with 52 siblings of the male patients. In males being treated with intensified chemotherapy but not with moderate-dose chemotherapy, the fertility rate was significantly lower.

Longhi et al.^12^ revealed that female age also has an impact on fertility. In osteosarcoma and Ewing’s sarcoma, patients of older age were a predisposing factor for infertility.

Several guidelines^4–9^ recommend that female and male patients with cancer should be counseled about the risk of infertility and the options for fertility preservation measures. Based on the available studies, patients can only be informed that chemotherapies used from1964 to 2012 do impose a clinical risk of infertility. However, it is not possible to provide accurate and age-related data.

This raises the question if the limited data on the fertility risk still apply to more recent chemotherapy protocols.

In Ewing’s sarcoma, ifosfamide was introduced in the early 1980s because of its milder myelotoxicity^31^ and therefore possibly lower gonadotoxicity, but the milder myelotoxicity allowed the introduction of high-dose chemotherapies, which would have neutralized such a putative lower gonadotoxic risk.

In osteosarcoma, alkylants are no longer part of the “standard” chemotherapy regimen. Therefore, the gonadotoxicity in more recent osteosarcoma chemotherapy protocols might be lower.

In postpubertal males, the deficit of data is clinically not that relevant as cryopreservation of sperm is easy, not very expensive, and can be performed within 1 day. In contrast, in prepubertal men and in females, this deficit is a major challenge. Freezing of testicular tissue in prepubertal boys is experimental^8,32^ and is only performed by a few clinics and therefore requires extensive logistics. Freezing of oocytes requires at least 2 weeks and freezing of ovarian tissue ½ to 1 week of lead time.^33,34^ These techniques are invasive and expensive and possibly require postponement of the chemotherapy, which might be a risk for the patients. This risk needs to be weighed against the potential success rate of the fertility preservation techniques. In males, the chance to father a baby using cryopreserved sperm is around 50%,^35^ but the chance is unknown for cryopreserved prepubertal testicular tissue. In females <35 years of age, the live birth rate is around 40% for oocytes vitrified before cancer therapies^36^ and around 30%–40% for cryopreserved ovarian tissue.^37,38^

Therefore, to improve infertility risk counseling and sharpen indications for fertility-preserving interventions, large studies are needed to acquire more recent, age-related, and sex-specific fertility data of high quality after osteosarcoma, Ewing’s sarcoma, and other cancer therapies. The collection of such data requires multicenter and multinational approaches to get a sufficient amount of data and to reflect the different treatment modalities applied around the world. Approaches such as the FertiTOX project (www.fertitox.com; Published study protocol: JMIR Res Protocol, published in 2024),^14^ which is organized by the network FertiPROTEKT (www.fertiprotekt.com), are a model for such a study. The project FertiTOX consists of three parts.

First, previously published studies are systematically analyzed regarding the gonadotoxicity of chemotherapies and radiotherapies in patients with cancer. This article belongs to this part.

Second, a prospective cohort study has been set up by approximately 60 centers in Germany, Switzerland, and Austria, and the following data will be collected: evaluation of ovarian function by analyzing AMH concentrations and testicular function by analyzing sperm parameters and total testosterone just before and around 1 year after gonadotoxic therapies (short-term fertility). Data collection started in December 1, 2023. A follow-up of these fertility parameters including the history of conceptions will be performed 5 and 10 years after gonadotoxic therapies (long-term fertility). In addition, the proportion of patients undergoing fertility-preserving methods, the satisfaction with the methods, and the amount of gametes and gonadal tissue and the children achieved by using the frozen material will be analyzed.

Third, the data will be merged to create the internet-based data platform FertiTOX. The data will be made available to any physicians and patients worldwide.

In conclusion, published data reveal a high variability of data regarding the risk of infertility in young female and male patients treated by chemotherapy for osteosarcoma and Ewing’s sarcoma. As some studies indicate a high and therefore clinically relevant infertility risk, female and male patients should be counseled about this risk and also about fertility preservation measures. This seems to be especially relevant in chemotherapy regimens containing alkylants. However, further prospective and large-scale studies are urgently needed to better calculate the fertility risk and to sharpen the indications for or against fertility preservation measures.
