# Supplementary material for: A Systematic Review of the Gonadotoxicity of Osteosarcoma and Ewing’s Sarcoma Chemotherapies in Postpubertal Females and Males
Source: J Adolesc Young Adult Oncol. 2024 Aug 2;13(4):597–606. doi: 10.1089/jayao.2023.0185 (PMC11322626; doi:10.1089/jayao.2023.0185)
Supplement: Supplementary Data S1 [file jayao.2023.0185_supplements1researchstrategy.pdf]

SUPPLEMENT S1

Ovid MEDLINE(R) ALL <1946 to present>

1 (chondrosarcoma* or chondro-sarcoma* or chrondroblastic sarcoma* or chondromucosarcoma* or chondromuco-sarcoma* or condrosarcoma* or condro-sarcoma).ti,ab,kf. 8890

2 exp Chondrosarcoma/ 7404

3 1 or 2 10725

4 (fibrosarcoma* or fibro-sarcoma* or fibroadenomasarcoma* or fibroadenoma-sarcoma* or fibroblastic sarcoma* or dermatofibrosarcoma* or dermatofibro-sarcoma* or neurofibrosarcoma* or neurofibro-sarcoma* or neuro-fibrosarcom*).ti,ab,kf.14642

5 exp Fibrosarcoma/ 13989

6 4 or 5 20294

7 (osteosarcoma* or osteo-sarcoma* or intracortical sarcoma* or osteogenic sarcoma* or osteoid sarcoma* or osteolytic sarcoma* or bone sarcoma* or bone cancer* or bone neoplasm* or ewing* sarcoma* or ewing* neoplasm* or ewing* tumo?r* or peripheral neuroectodermal tumo?r* or pnet* or mpnet*).ti,ab,kf. 44136

8 exp Osteosarcoma/ 31306

9 7 or 8 50724

10 (chemotherap* or chemo-therap* or chemoradiotherap* or chemo-radiotherap* or adjuvant drug therap* or carcinochemotherap* or antineoplastic agent* or anti-neosplastic agent* or antineoplastic drug* or anti-neoplastic drug* or antitumo?r agent* or anti-tumo?r agent* or antitumo?r drug* or anti-tumo?r drug* or anticancer* agent* or anti-cancer* agent* or anticancer* drug* or anti-cancer drug* or anticarcinogen* or anti-carcinogen* or anticancerogen* or anti-cancerogen* or ((cancer* or tumo?r* or neoplasm*) adj3 treat*)).ti,ab,kf. 780118

11 exp Antineoplastic Agents/ 1210399

12 exp Combined Modality Therapy/ 290373

13 exp Antineoplastic Combined Chemotherapy Protocols/ 155845

14 exp chemotherapy, adjuvant/ 45284

15 exp Antineoplastic Protocols/ 156394

16 exp chemoradiotherapy/ or radioimmunotherapy/ or exp radiotherapy, adjuvant/ 45527

17 10 or 11 or 12 or 13 or 14 or 15 or 161849180

18 (Radio-therap* or radiotherap* or radiationtherap* or chemoradiotherap* or radiochemotherap* or protontherap* or radiosurg* or radio-surg* or irradiation* or x-ray-therap* or therap* radiolog* or IMRT* or IORT* or radioimmunotherap* or radio-immuno-therap* or ((radiat* or irradiat* or radioisotope* or radio-isotope* or chemoradio or chemo-radio or radiochemo or radio-chemo or proton or x-ray or xray) adj2 (therap* or oncolog* or brachytherap* or brachy-therap*))).ti,ab,kf. 493312

19 exp Radiotherapy/ 202968

20 18 or 19 549877

21 (fertili#ation* or fertility or fertile or fecundity or fecundability or subfecundity or sub-fecundity or infecundity or infertility or sterility or subfertility or subfertile or sub-fertility or sub-fertile or anovularit* or gonad* or reproductive organ* or reproduction* or gamete-producing gland* or ovarian reserve* or ovary or ovaries or ovarian follicle* or oogenesis or oocyte* or amenorrhea* or premature menopaus* or early menopaus* or climacterium pr?ecox or Gonadotropin* or AMH or Anti-Mu?llerian Hormone* or Antimu?llerian Hormone* or Anti-Mu?llerian Factor* or Mu?llerian Inhibiting Hormone* or mu?llerian inhibitor* or FSH or Follicle Stimulating Hormone* or Folliculostimulating Hormone* or Follitropin or FSH-releasing hormone or LH-FSH or testis or testes or testicle* or spermatogenes* or sperm* or semen or gametogenes* or hypogonadism* or hypo-gonadism* or "reproductive system*" or azoospermia* or spermatozoon*).ti,ab,kf. 743916

22 exp Fertility/ 44916

23 exp Infertility/ 71753

24 exp Gonads/ 172782

25 Amenorrhea/ 10080

26 anovulation/ or menopause, premature/ 3434

27 Reproduction/ 63956

28 Gametogenesis/ 1421

29 Spermatozoa/ 62087

30 21 or 22 or 23 or 24 or 25 or 26 or 27 or 28 or 29 835434

31 17 or 20 2183241

32 3 or 6 or 9 77376

33 30 and 31 and 32 354

34 exp animals/ not humans.sh. 5035392

35 33 not 34 317

36 limit 35 to yr="2000-current" 215

Ovid Embase <1974 to present>

1 (chondrosarcoma* or chondro-sarcoma* or chrondroblastic sarcoma* or chondromucosarcoma* or chondromuco-sarcoma* or condrosarcoma* or condro-sarcoma).ti,ab,kf. 10735

2 exp chondrosarcoma/ 12583

3 1 or 2 14702

4 (fibrosarcoma* or fibro-sarcoma* or fibroadenomasarcoma* or fibroadenoma-sarcoma* or fibroblastic sarcoma* or dermatofibrosarcoma* or dermatofibro-sarcoma* or neurofibrosarcoma* or neurofibro-sarcoma* or neuro-fibrosarcom*).ti,ab,kf.16432

5 exp fibrosarcoma/ 17953

6 4 or 5 22809

7 (osteosarcoma* or osteo-sarcoma* or osteogenic sarcoma* or intracortical sarcoma* or or osteoid sarcoma* or osteolytic sarcoma* or bone sarcoma* or bone cancer* or bone neoplasm* or ewing* sarcoma* or ewing* neoplasm* or ewing* tumo?r* or peripheral neuroectodermal tumo?r* or pnet* or mpnet*).ti,ab,kf. 56621

8 exp osteosarcoma/ 39433

9 7 or 8 67711

10 3 or 6 or 9 98018

11 (chemotherap* or chemo-therap* or chemoradiotherap* or chemo-radiotherap* or adjuvant drug therap* or carcinochemotherap* or antineoplastic agent* or anti-neosplastic agent* or antineoplastic drug* or anti-neoplastic drug* or antitumo?r agent* or anti-tumo?r agent* or antitumo?r drug* or anti-tumo?r drug* or anticancer* agent* or anti-cancer* agent* or anticancer* drug* or anti-cancer drug* or anticarcinogen* or anti-carcinogen* or anticancerogen* or anti-cancerogen* or ((cancer* or tumo?r* or neoplasm*) adj3 treat*)).ti,ab,kf. 1170677

12 exp antineoplastic agent/ or exp multimodality cancer therapy/ or exp cancer chemotherapy/ or exp antineoplastic protocol/ 2751130

13 (Radio-therap* or radiotherap* or radiationtherap* or chemoradiotherap* or radiochemotherap* or protontherap* or radiosurg* or radio-surg* or irradiation* or x-ray-therap* or therap* radiolog* or IMRT* or IORT* or radioimmunotherap* or radio-immuno-therap* or ((radiat* or irradiat* or radioisotope* or radio-isotope* or chemoradio or chemo-radio or radiochemo or radio-chemo or proton or x-ray or xray) adj2 (therap* or oncolog* or brachytherap* or brachy-therap*))).ti,ab,kf. 672319

14 exp cancer radiotherapy/ 302022

15 11 or 12 or 13 or 14 3562748

16 (fertili#ation* or fertility or fertile or fecundity or fecundability or subfecundity or sub-fecundity or infecundity or infertility or sterility or subfertility or subfertile or sub-fertility or sub-fertile or anovularit* or gonad* or reproductive organ* or reproduction* or gamete-producing gland* or ovarian reserve* or ovary or ovaries or ovarian follicle* or oogenesis or oocyte* or amenorrhea* or premature menopaus* or early menopaus* or climacterium pr?ecox or Gonadotropin* or AMH or Anti-Mu?llerian Hormone* or Antimu?llerian Hormone* or Anti-Mu?llerian Factor* or Mu?llerian Inhibiting Hormone* or mu?llerian inhibitor* or FSH or Follicle Stimulating Hormone* or Folliculostimulating Hormone* or Follitropin or FSH-releasing hormone* or LH-FSH or testis or testes or testicle* or spermatogenes* or sperm* or semen or gametogenes* or hypogonadism* or hypo-gonadism* or "reproductive system*" or azoospermia* or spermatozoon*).ti,ab,kf. 833144

17 exp fertility/ or exp infertility/ or exp semen analysis/ or exp gonad/ or exp amenorrhea/ or exp early menopause/ or reproduction/ or gametogenesis/ or spermatozoon/ 548671

18 16 or 17 973906

19 10 and 15 and 18 897

20 (exp animal/ or exp invertebrate/ or nonhuman/ or animal experiment/ or animal tissue/ or animal model/ or exp plant/ or exp fungus/) not (exp human/ or human tissue/) 7411353

21 19 not 20 828

22 limit 21 to yr="2000-current" 719

Cochrane Library <1996 to present>

#1 (chondrosarcoma* or chondro-sarcoma* or "chrondroblastic sarcoma*" or chondromucosarcoma* or chondromuco-sarcoma* or condrosarcoma* or condro-sarcoma):ti,ab,kw (Word variations have been searched) 97

#2 (fibrosarcoma* or fibro-sarcoma* or fibroadenomasarcoma* or fibroadenoma-sarcoma* or "fibroblastic sarcoma*" or dermatofibrosarcoma* or dermatofibro-sarcoma* or neurofibrosarcoma* or neurofibro-sarcoma* or neuro-fibrosarcom*):ti,ab,kw 75

#3 (osteosarcoma* or osteo-sarcoma* or "intracortical sarcoma*" or "osteogenic sarcoma*" or "osteoid sarcoma*" or "osteolytic sarcoma*" or "bone sarcoma*" or "bone cancer*" or "bone neoplasm*" or "ewing* sarcoma*" or "ewing* neoplasm*" or "ewing* tumor*" or "ewing* tumo?r*" or "peripheral neuroectodermal tumo?r*" or "peripheral neuroectodermal tumor*" or pnet* or mpnet* or sarcoma*):ti,ab,kw 3630

#4 (chemotherap* or chemo-therap* or chemoradiotherap* or chemo-radiotherap* or "adjuvant drug therap*" or carcinochemotherap* or "antineoplastic agent*" or "anti-neosplastic agent*" or "antineoplastic drug*" or "anti-neoplastic drug*" or "antitumo?r agent*" or "antitumor agent*" or "anti-tumo?r agent*" or "anti-tumor agent*" or "antitumo?r drug*" or "antitumor drug*" or "anti-tumo?r drug*" or "anti-tumor drug*" or "anticancer* agent*" or "anti-cancer* agent*" or "anticancer* drug*" or "anti-cancer drug*" or anticarcinogen* or anti-carcinogen* or anticancerogen* or anti-cancerogen*):ti,ab,kw or ((cancer* or tumo?r* or tumor* or neoplasm*) NEAR/3 treat*):ti,ab,kw 105082

#5 (Radio-therap* or radiotherap* or radiationtherap* or chemoradiotherap* or radiochemotherap* or protontherap* or radiosurg* or radio-surg* or irradiation* or x-ray-therap* or therap* radiolog* or IMRT or IORT or radioimmunotherapy* or radio-immuno-therap* or ((radiat* or radioisotope* or radio-isotope* or chemoradio or chemo-radio or radiochemo or radio-chemo or proton* or x-ray* or xray*) near/2 (therap* or oncolog* or brachytherap* or brachy-therap*))):ti,ab,kw 54290

#6 (fertilization* or fertilisation* or fertility or fertile or fecundity or fecundability or subfecundity or sub-fecundity or infecundity or infertility or sterility or subfertility or subfertile or sub-fertility or sub-fertile or anovularit* or gonad* or "reproductive organ*" or reproduction* or "gamete-producing gland*" or "ovarian reserve*" or ovary or ovaries or "ovarian follicle*" or oogenesis or oocyte* or

amenorrhea* or "premature menopaus*" or "early menopaus*" or "climacterium pr?ecox" or "climacterium precox" or Gonadotropin* or AMH or "Anti-Mu?llerian Hormone*" or "Antimu?llerian Hormone*" or "Anti-Mu?llerian Factor*" or "Mu?llerian Inhibiting Hormone*" or "mu?llerian inhibitor*" or "Anti-Mullerian Hormone*" or "Antimullerian Hormone*" or "Anti-Mullerian Factor*" or "Mullerian Inhibiting Hormone*" or "mullerian inhibitor*" or FSH or "Follicle Stimulating Hormone*" or "Folliculostimulating Hormone*" or Follitropin or "FSH-releasing hormone*" or LH-FSH or testis or testes or testicle* or spermatogenes* or sperm* or semen or gametogenes* or hypogonadism* or hypo-gonadism* or "reproductive system*" or azoospermia* or spermatozoon*):ti,ab,kw 38934

#7 #1 or #2 or #3 3683

#8 #4 or #5 133032

#9 #6 and #7 and #8 with Cochrane Library publication date Between Jan 2000 and Sep 2022 92
